# Abundance and Diversity of Dung Beetles (Coleoptera: Scarabaeoidea) as Affected by Grazing Management in the Nebraska Sandhills Ecosystem

**DOI:** 10.1093/ee/nvaa130

**Published:** 2020-11-13

**Authors:** Patrick M Wagner, Gandura Omar Abagandura, Martha Mamo, Thomas Weissling, Ana Wingeyer, Jeffrey D Bradshaw

**Affiliations:** 1 Department of Entomology, University of Nebraska-Lincoln, Lincoln, NE; 2 Agronomy, Horticulture & Plant Science, South Dakota State University, Brookings, SD; 3 Agronomy and Horticulture, University of Nebraska-Lincoln, Lincoln, NE; 4 Instituto Nacional de Tecnología Agropecuaria, Ruta Oro Verde Entre Ríos, Argentina

**Keywords:** beetle, dung, grazing, rangeland

## Abstract

Dung beetles (Coleoptera: Scarabaeoidea) serve a significant role in regulating ecosystem services on rangelands. However, the influence of grazing management on dung beetle communities remains largely unknown. The purpose of this study was to investigate dung beetle abundance and diversity throughout the grazing season in the Nebraska Sandhills Ecoregion. Grazing treatments included: continuous grazing (CONT), low-stocking rotational grazing (LSR), high-stocking rotational grazing (HSR), and no grazing (NG). The abundance and diversity of dung beetles were measured in the 2014 and 2015 grazing seasons using dung-baited pitfall traps. Dung beetle abundance for each grazing treatment was characterized through four indices: peak abundance, species richness, Simpson’s diversity index, and Simpson’s evenness. A total of 4,192 dung beetles were collected through both years of trapping in this study. Peak abundance and species richness were greater in grazed treatments when compared to NG in both years. Peak abundance in the HSR was 200% (2014) and 120% (2015) higher than in the LSR. Species richness in the HSR was 70% (2014) and 61% (2015) higher than in the LSR, and 89% (2014) and 133% (2015) higher than in CONT. Simpson’s diversity index was lower in the NG and CONT treatments when compared to the LSR or HSR treatments for both years. We conclude that rotational grazing, regardless of stocking density, promoted dung beetle abundance and diversity within the Nebraska Sandhills Ecoregion.

Dung beetles (Coleoptera: Scarabaeoidea) serve an important role in the function of many ecosystems (Perrin et al. 2020). Dung beetles scavenge dung from the soil surface and transport it underground where they then use it as a food resource (Halffter and Matthews 1966, Simmons and Ridsdill-Smith 2011, Nunes et al. 2018). A number of studies have reported that dung beetles can play important roles in nutrient cycling, greenhouse gas mitigation, parasite suppression, and overall trophic regulation (Bang et al. 2005, Yamada et al. 2007, Nichols et al. 2008, Penttilä et al. 2013, Santos-Heredia et al. 2018, Evans et al., 2019a). In addition, dung beetle activity can increase soil nutrients at the soil surface by incorporating nutrients from the dung into the soil (Evans et al. 2019b). Therefore, dung beetle activity is recognized as being very important for ranch management by promoting and maintaining healthy cattle-grazed rangeland ecosystems (Aarons et al. 2009, Menéndez et al. 2016). The relationships between dung beetle activity and these important ecological functions demonstrate why dung beetles are important for the promotion and maintenance of healthy cattle-grazed ecosystems (Perrin et al. 2020). The ecosystem services provided by dung beetles are estimated to be over $380 million annually in the United States (Losey and Vaughan 2006). However, the value of the services provided by dung beetles is greatly affected by their biodiversity, with less diverse communities providing fewer ecosystem functions (Spaak et al. 2017). Manning et al. (2017) determined higher dung beetle species richness had greater pasture productivity when compared to lower dung beetle species richness.

Therefore, reports of declining species in the last decade due to habitat fragmentation could result in reduced effectiveness of dung beetles within rangeland ecosystems (Hutton and Giller 2003, Filgueiras et al. 2011, Slade et al. 2016, Perrin et al. 2020). Due to these findings, an increased number of ranchers are interested in conserving and promoting dung beetles on their grasslands (Hutton and Giller 2003, Slade et al. 2016). Intensifying grazing practices that promote dung beetle activity could be beneficial for ranchers by improving the ecosystem services they provide on rangeland (Verdú et al. 2007, Correa et al. 2019).

In Nebraska, there are over nine million hectares of rangeland and pasture that are primarily used for grazing (Nebraska Department of Agriculture 2016). On average, cattle produce 8–12 dung pats per day (Bornemissza 1960). This indicates that a single cow may foul approximately 0.4–0.96 m^2^ of grassland per day (Fincher 1981, Yoshitoshi et al. 2016). However, grazing management can have an impact on the dung production. Grazing management is performed on rangeland in numerous ways with two of the most common practices being continuous and rotational grazing (Zhou et al. 2019). Continuous grazing involves grazing cattle at low stocking densities in a single, open pasture for the duration of the grazing season. This is to ensure that there is enough forage to last the entire season (Holechek et al. 2011). Rotational grazing involves splitting pastures into multiple paddocks with cattle being rotated through each paddock as available forage becomes depleted (de la Motte et al. 2018). Rotational grazing allows higher stocking densities than that of continuous grazing. High-stocking rotational grazing (HSR grazing) involves the rotation of high densities of cattle (~500 AU/ha) through small paddocks for short time durations of 1 d or less (Gompert 2009, Thomas 2012). The goal behind high-stocking rotational grazing is to improve pasture productivity by increasing cattle grazing efficiency (Aarons et al. 2009, Moir et al. 2010).

Several studies have reported that grazing can positively influence dung beetle occurrence and diversity (Hutton and Giller 2003, Numa et al. 2010). Buse et al. (2015) reported that grazing continuity had positive effects on total species richness. However, the impact that specific grazing practices have on dung beetle occurrence and diversity remains largely unknown (Lee and Wall 2006, Verdú et al. 2007). Whipple (2011) collected over six times the number of dung beetles and twice the number of dung beetle species in rotationally grazed paddocks relative to continuous grazing. The number of collected dung beetles was greater in high grazing intensity compared to low grazing intensity on the herbaceous vegetation due to the fact that high grazing intensity can increase dung quantity (Perrin et al. 2020).

Although several factors could influence dung beetle abundance and diversity between grazing practices, it is hypothesized that higher stocking density would increase concentration of dung pats deposited per paddock in a short time, thus increasing the abundance and species diversity of dung beetles. The purpose of this study was to quantify the abundance and diversity of dung beetles under different grazing treatments.

## Materials and Methods

### Study Site Description

This study was conducted in 2014 and 2015 on ~25 ha of grassland at the University of Nebraska-Lincoln’s Barta Brothers Ranch (BBR, 42°13′N; 99°38′W) in the northeastern Sandhills Ecoregion of Nebraska. Situated above the Ogallala Aquifer, this ecoregion is composed of grass-covered sand dunes and sub-irrigated (aquifer-fed) meadows with numerous lakes and wetlands spread throughout (Ahlbrandt and Fryberger 1980, Rundquist 1983). According to Lindsey (2016) and Guretzky et al. (2020), perennial, exotic cool-season grasses, including timothy (*Phleum pratense* L. (Poales: Poaceae)), quackgrass (*Elymus repens* Gould (Poales: Poaceae)), red-top (*Agrostis stolonifera* L. (Poales: Poaceae)), and Kentucky bluegrass (*Poa pratensis* L. (Poales: Poaceae)), dominated the meadow; however, perennial, native warm-season grasses, sedges, and rushes were common. Soils are sandy to fine sandy loam texture. Soil organic matter content ranged between 14 and 33 mg/g at the 0- to 10-cm depth and between 4 and 9 mg/g at the 10- to 20-cm depth (Evans et al., 2019b). Meadows are seasonally wet in early to late spring due to the rising water table. The growing season lasts ~ 150 d with annual precipitation ranging from 430 to 580 mm and temperature averages of ~10°C.

To provide observations and descriptive statistics for the dung beetle community in the region, a dung beetle survey was completed at two private ranches in the northeastern Sandhills. The private ranches were the Brown County Ranch (BCR) and the Rock County Ranch (RCR). The study area at the BCR (42°19′N; 100°4′W) was ~303 ha of sub-irrigated meadow and ~364 ha of sandy upland. The study area at the RCR (42°29′N; 99°20′W) was ~32 ha and consisted of sub-irrigated meadow. The distance between BBR and each private ranch is approximately 37 km. The distance between the private ranches is approximately 63 km.

### Grazing Management

Although grazing occurred on all of the ranches in 2014 and 2015, the effect of grazing on dung beetle abundance and diversity was only evaluated at the BBR because grazing management treatments at BCR and RCR were not replicated. Information about the grazing management at these two private ranches can be seen in Supp Table S1 (online only). For the BBR in 2014 and 2015, grazing began in early June when cattle were placed onto pasture and continued until cattle were removed in late August/September. The ranch was grazed by a separate cattle herd (yearling steers) for the duration of the grazing seasons. Cattle were tagged with insecticidal ear tags to prevent flies; however, they were not treated with any other insecticides (ivermectins, cydectins, etc.) prior to being placed onto pasture. The presence of insecticidal ear tags was not a concern in this study as they prove to have minimal impact on dung beetle populations (Schreiber et al. 1987, Bertone et al. 2004).

Five grazing management treatments were applied at BBR in a randomized complete block design with two replicates: continuous (CONT), low-stocking rotational grazing which consisted of once-over (LSR once-over) and twice-over (LSR twice-over) rotational treatments, high-stocking density rotational grazing (HSR), and no grazing (NG) as a control. Cattle stocking rates were the same across all treatment pastures. However, the rotational pastures were divided into smaller paddocks and had different stocking densities depending on the treatment. In the CONT, cattle were grazed at low stocking densities (<1 AU/ha) and were kept in a single open pasture for the duration of the grazing season. For the LSR once-over, cattle were grazed at low stocking densities (~20 AU/ha) and were moved to a new paddock every 3–4 wk. These cattle grazed each paddock once each season. In the LSR twice-over, cattle were also grazed at low stocking densities (~20 AU/ha), but were moved to a new paddock every 1–2 wk. Thus, these cattle grazed each paddock twice each season. For the HSR, cattle were grazed at ultra-high stocking densities (~500 AU/ha) and were moved to a new paddock two times per day. These cattle grazed each paddock once each season. Lastly, the NG had no cattle present throughout the grazing season. A detailed explanation of the grazing treatments at the BBR can be found in Table 1.

**Table 1.** Grazing managements, abbreviations, stocking densities, number of traps, and area per trap for Barta Brothers Ranch in the Nebraska Sandhills in 2014 and 2015

Grazing management	Grazing abbreviation	Stocking density per paddock (AU/ha)	Number of traps	Hectare per trap
No graze	NG	0	4	0.4
Continuous grazing	CONT	<1	4	0.4
Low-stocking rotational grazing with once-over	LSR once-over	~20	8	0.5
Low-stocking rotational grazing with twice-over	LSR twice-over	~20	8	0.5
High-stocking rotational grazing	HSR	~500	36	0.4

### Sampling

**Fig. 1.**
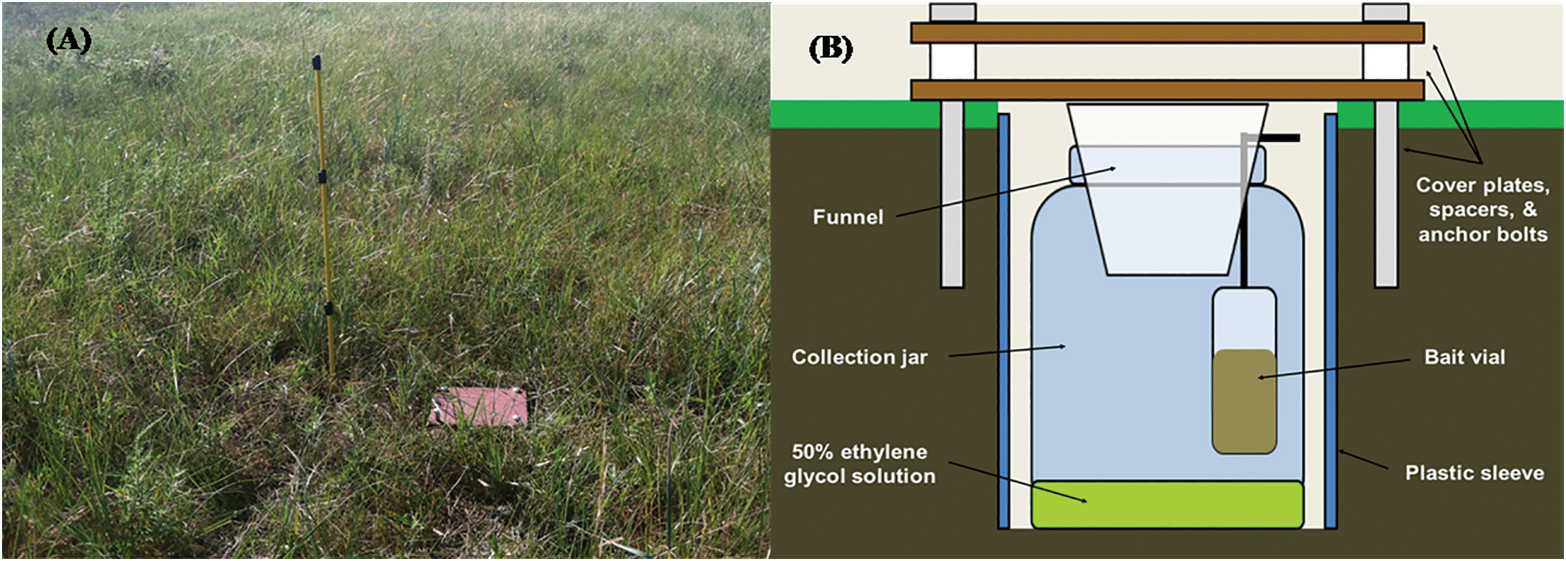
Photograph of a pitfall trap (A) and diagram of the pitfall trap design (B) that was used to measure dung beetle activity in grazing treatments during the 2014 and 2015 grazing seasons.

Sampling was conducted in all ranches using pitfall traps (Fig. 1A) from June to August in 2014 and 2015 to monitor the abundance and diversity of dung beetles on pastures. Pitfall traps were placed in each grazing treatment throughout each study pasture and spaced ~50 m apart to ensure that no interference occurred (Larsen and Forsyth 2005). Traps were designed similar to Ratcliffe (2013); however, they were modified using 500 ml Nalgene jars and steel cover plates. Cover plates were used to prevent cattle from stepping into the traps, and the plates were separated ~2.5 cm apart with PVC spacers (Fig. 1B). Each trap was baited with a 20-ml vial containing approximately 10–20 ml of homogenized primate dung from chimpanzees fed on a standard diet. Chimpanzee dung was used as bait on the basis that dung beetles exhibit higher attraction to primate dung compared to that of other animals (Whipple and Hoback 2012). For a killing agent, Nalgene jars were filled with approximately 50–100 ml of a 50% ethylene glycol/water solution.

At BBR, 36 traps were present in the HSR treatment, 8 traps were present in the LSR once-over and LSR twice-over treatments, and 4 traps were present in the CONT and NG treatments. The increased number of traps in the HSR and LSR treatments were to account for the fact that cattle were rotated to different paddocks within the pastures throughout each season. Only the traps that were in the same paddocks as the cattle each week were used for the statistical analysis. For all treatments, this equaled to approximately 0.4–0.5 ha per trap (Table 1). For BCR meadow and upland, there were 30 and 15 traps both years, respectively. For RCR, there were 18 traps in 2014.

**Fig. 2.**
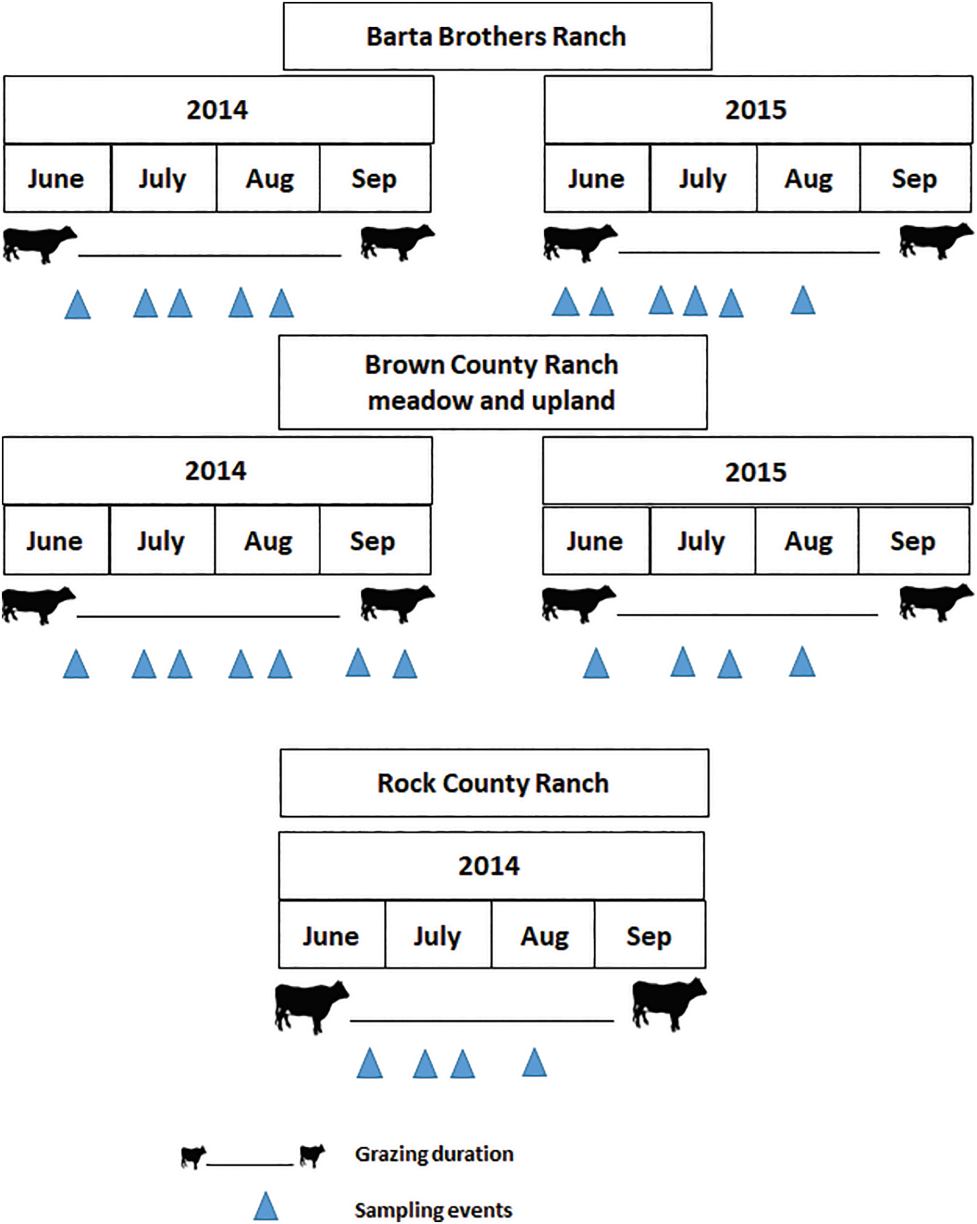
Timeline of grazing and dung beetles sampling performed at the Barta Brothers Ranch, Brown County Ranch, and Rock County Ranch in 2014 and 2015 in the Nebraska Sandhills.

Following similar methods to Whipple and Hoback (2012), the traps were baited for 7-d intervals within 14-d periods. This allowed the traps to be temporarily sealed up in case of heavy rain or a flooding event. Traps were collected at the end of each 14-d period and bait vials were replaced with fresh dung for the following period. Sampling time in all ranches can be seen in Fig. 2. For BBR, 2014 sampling began on June 25 and concluded on August 20 (five sampling events), and 2015 sampling began on June 2 and concluded on August 11 (six sampling events). For BCR meadow and upland, 2014 sampling occurred from June 24 to September 18 (seven sampling events for each position) and 2015 sampling occurred from June 17 to August 12 (four events for each position). For RCR, 2014 sampling occurred from June 30 to August 11 (four events) and no sampling occurred in 2015 due to a change in ownership of the land. Following collection, samples were taken to the laboratory where dung beetles were counted and identified to species according to Ratcliffe and Paulsen (2008). Dung beetle species verification was conducted with the assistance of the collections manager at the University of Nebraska State Museum. A voucher collection of dung beetle species was constructed for this study and is housed at the University of Nebraska State Museum in Lincoln, NE.

### Adequacy of Sampling

To determine the adequacy of sampling, species accumulation curves for all ranches were generated using EstimateS version 9.1.0 (Colwell 2013). Interpolation was used to show how species richness increased per sample up to the number of samples that were empirically collected. Interpolation uses the rarefaction technique to estimate expected species richness from a random subsampling of the data (Gotelli 2008). An estimation of the asymptotic species was then generated with extrapolation to show how much more sampling would need to take place before additional species would be collected. Accumulation curves were generated from all collected samples for each study location in 2014 and 2015 combined. For the direct statistical comparison of the accumulation curves, we calculated the number of species observed ± the 95% CIs.

### Statistical Procedures

Dung beetle activity for each grazing treatment at the BBR was characterized through four indices: peak abundance per trap, species richness, Simpson’s diversity index, and Simpson’s evenness. Peak abundance was defined as the maximum number of dung beetles recovered per trap per period for each grazing treatment when cattle were present in the paddock. This was used to counteract the absence of cattle in paddocks that were not being grazed in the rotational grazing treatments. Species richness, or number of species, was expressed as the total number of species that were captured in each grazing treatment. Simpson’s diversity index (*D*) quantifies the diversity in a habitat by accounting for species richness as well as the relative abundance of each species in a sample (Magurran and McGill 2011) by the following equation:


$$\begin{array}{l} D\;=\;\Sigma {p_{i}}^{2} \end{array}$$ (1)

where *p*_*i*_ represents the proportion of abundance for species *i*. Simpson’s diversity index can be summarized as, ‘the probability that two individuals drawn at random from an infinite community would belong to the same species’. The reciprocal of Simpson’s diversity index was used to determine dung beetle diversity across each grazing treatment. It ranges on a scale from 1 to the maximum number of species collected, with higher values signifying more diversity in a sample. Lastly, Simpson’s evenness, a measure of the relative abundance of species in a community, was estimated as follows (Magurran and McGill 2011):


$$\begin{array}{l} E\;=\;\left(1/D\right)/S \end{array}$$ (2)

where *1*/*D* represents the reciprocal of Simpson’s diversity index, and *S* represents the total number of species in the community. Simpson’s evenness ranges on a scale from 0–1, with 0 indicating maximum unevenness and 1 indicating perfect evenness.

All data were analyzed using a mixed model (PROC MIXED procedure) in SAS (2013) statistical software version 9.4, with grazing management and year being considered as fixed factors and replications as a random factor for each ranch. Significantly different treatment means were separated using a Tukey’s HSD mean comparison test with an α = 0.05 significance level.

## Results

### Dung Beetle Collection Totals

The overall total number of dung beetles collected through both years of this study in all ranches was 4,192. Percent abundance for all species collected at each ranch is presented in Table 2. Across all grazing treatments in 2014, a total of 760 dung beetles were collected at the BBR, 394 in the meadow and 67 in the upland at the BCR, and 564 at the RCR, with a grand total of 1,785. In 2015, a total of 1,441 were collected at the BBR and 538 in the meadow and 428 in the upland at the BCR, with a grand total of 2,407. The species composition varied across ranches, with *Onthophagus* spp. Latreille being the most dominant and consistent dung beetles found across all locations in 2014 and 2015 (Table 2). The total number of dung beetle species collected was 12 for the BBR both years, 10 (2014) and 9 (2015) in the BCR meadow, 7 (2014) and 12 (2015) in the BCR upland, and 9 in the RCR (Table 2).

**Table 2.** Percent abundance of dung beetle (Coleoptera: Scarabaeoidea) species collected at Barta Brothers Ranch (BBR), Brown County Ranch (BCR) in meadow and upland, and Rock County Ranch (RCR) in 2014 and 2015 in the Nebraska Sandhills

Species	2014	2015	Average
BBR			
*Onthophagus hecate (Panzer)*	36.58	52.05	44.32
*Diapterna pinguella* (Brown)	41.45	23.87	32.66
*Onthophagus pennsylvanicus* Harold	5.53	13.95	9.74
*Aphodius rusicola* Haldeman	8.82	1.53	5.18
*Ataenius spretulus* (Haldeman)	5.13	3.75	4.44
*Onthophagus orpheus pseudorpheus* Howden and Cartwright	0.92	1.87	1.39
*Aphodius fimetarius* (Linnaeus)	0.53	1.04	0.79
*Aphodius rubeolus* Palisot de Beauvois	0.39	0.76	0.58
*Aphodius haemorrhoidalis* (Linnaeus)	0.26	0.76	0.51
*Geotrupes opacus* Haldeman	−	0.21	0.11
*Melanocanthon nigricornis* (Say)	−	0.14	0.07
*Aphodius gordoni* Ratcliffe	0.13	−	0.06
*Ataenius imbricatus* (Melsheimer)	0.13	−	0.06
*Canthon pilularius* (Linnaeus)	0.13	−	0.06
*Bolbocerosoma bruneri* Dawson and McColloch	−	0.07	0.03
BCR meadow			
*Diapterna pinguella* (Brown)	39.60	42.75	41.18
*Onthophagus hecate* (Panzer)	44.16	36.62	40.39
*Onthophagus pennsylvanicus* Harold	3.81	14.31	9.06
*Aphodius rusicola* Haldeman	9.90	1.49	5.69
*Melanocanthon nigricornis* (Say)	0.76	1.67	1.22
*Geotrupes opacus* Haldeman	−	2.04	1.02
*Ataenius spretulus* (Haldeman)	0.51	0.74	0.63
*Copris fricator* (Fabricius)	0.51	0.19	0.35
*Aphodius rubeolus* Palisot de Beauvois	0.25	0.19	0.22
*Odenteus filicornis* (Say)	0.25	−	0.12
*Onthophagus orpheus pseudorpheus* Howden and Cartwright	0.25	−	0.12
BCR upland			
*Onthophagus pennsylvanicus* Harold	28.36	43.46	35.91
*Onthophagus hecate* (Panzer)	32.84	35.05	33.95
*Canthon ebenus* (Say)	29.85	7.01	18.43
*Geotrupes opacus* Haldeman	−	6.54	3.27
*Melanocanthon nigricornis* (Say)	4.48	1.64	3.06
*Onthophagus orpheus pseudorpheus* Howden and Cartwright	1.49	1.87	1.68
*Aphodius rubeolus* Palisot de Beauvois	1.49	1.64	1.57
*Aphodius rusicola* Haldeman	1.49	0.23	0.86
*Canthon pilularius* (Linnaeus)	−	1.17	0.59
*Aphodius haemorrhoidalis* (Linnaeus)	−	0.93	0.46
*Copris fricator* (Fabricius)	−	0.23	0.11
*Phanaeus vindex* MacLeay	−	0.23	0.11
RCR			
*Onthophagus hecate* (Panzer)	57.09		
*Ataenius spretulus* (Haldeman)	13.12		
*Diapterna pinguella* (Brown)	13.12		
*Aphodius rusicola* Haldeman	10.99		
*Onthophagus pennylvanicus* Harold	3.01		
*Aphodius fimetarius* (Linnaeus)	0.71		
*Bolbocerosoma bruneri *Dawson and McColloch	0.71		
*Aphodius rubeolus* Palisot de Beauvois	0.35		
*Aphodius erraticus* (Linnaeus)	0.18		

### Species Accumulation Curves

**Fig. 3.**
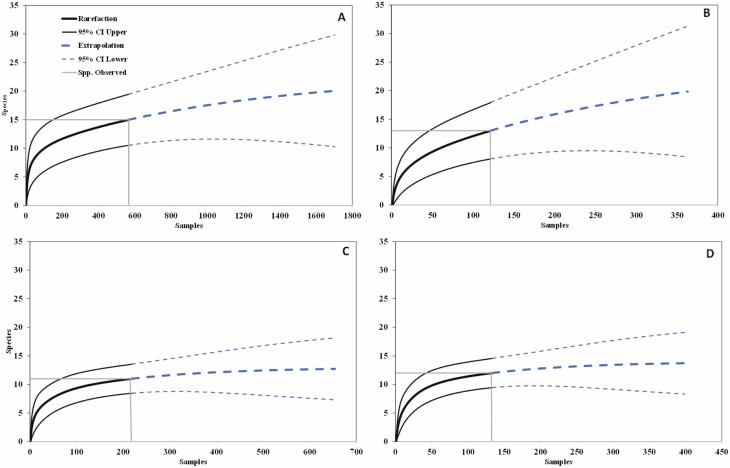
Accumulation curves of dung beetle (Coleoptera: Scarabaeoidea) species that were collected at the Barta Brothers Ranch (A), Rock County Ranch (B), and Brown County Ranch meadow (C) and upland (D) in 2014 and 2015. Interpolation (rarefaction) and extrapolation with 95% CIs. The gray line represents the species observed.

The generated accumulation curves revealed that the number of samples collected would need to significantly increase before new species could be observed. At the BBR, it is estimated that doubled sampling efforts would only result in the collection of three additional species (Fig. 3A). At RCR (2014 only), doubled sampling efforts would result in the collection of four additional species (Fig. 3B). In both the meadow and upland pastures at the BCR, doubled sampling efforts would only result in the collection of one additional species (Fig. 3C and D).

### Effect of Grazing on Dung Beetle Abundance and Diversity at the Barta Brothers Ranch

#### Peak Abundance

**Fig. 4.**
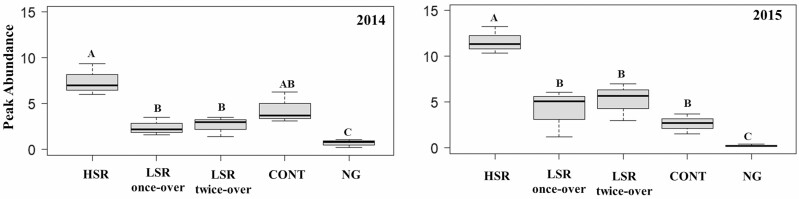
Mean peak abundance of dung beetles (Coleoptera: Scarabaeoidea) collected in 2014 and 2015 at Barta Brothers Ranch. Treatments are high-stocking rotational grazing (HSR), low-stocking rotational grazing with once-over (LSR once-over) and twice-over (LSR twice-over), continuous grazing (CONT), and no grazing (NG). Letters indicate significance in treatments (*P* < 0.05). Means with the same letters are not significantly different. Vertical bars indicate SEs of the means.

Average peak dung beetle abundance under different grazing treatments at the BBR is shown in Fig. 4. The average peak dung beetle abundances on grazed treatments were consistently higher than the NG during 2014 and 2015 (Fig. 4). In 2014, the HSR was 218% and 200% higher than the LSR once-over (*F*_1,86_ = 10.49, *P* = 0.0017) and LSR twice-over (*F*_1,86_ = 9.47, *P* = 0.0028), respectively, but similar to the CONT (*P* = 0.0831) (Fig. 4). In 2015, results indicated significantly higher peak abundance in the HSR compared to the LSR once-over (265%, *F*_1,104_ = 14.87, *P* = 0.0002), LSR twice-over (120%, *F*_1,104_ = 6.02, *P* = 0.0158), and the CONT (328%, *F*_1,104_ = 16.38, *P* < 0.0001) (Fig. 4).

#### Species Richness

**Fig. 5.**
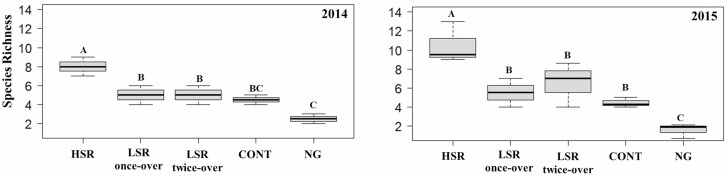
Species richness of dung beetles (Coleoptera: Scarabaeoidea) collected at the Barta Brothers Ranch in 2014 and 2015. Treatments are high-stocking rotational grazing (HSR), low-stocking rotational grazing with once-over (LSR once-over) and twice-over (LSR twice-over), continuous grazing (CONT), and no grazing (NG). Letters indicate significance in treatments (*P* < 0.05). Means with the same letters are not significantly different. Vertical bars indicate SEs of the means.

Average species richness under different grazing treatments at the BBR is shown in Fig. 5. All treatments over both years were significantly higher than the NG treatment, except for the CONT in 2014 (Fig. 5). In both years, species richness in the HSR was significantly higher than the LSR once-over (70%, *F*_1,5_ = 17.53, *P* = 0.0086 in 2014 and 91%, *F*_1,5_ = 19.26, *P* = 0.0071 in 2015), the LSR twice-over (70%, *F*_1,5_ = 17.53, *P* = 0.0086 in 2014 and 61%, *F*_1,5_ = 12.32, *P* = 0.0171 in 2015), and the CONT (89%, *F*_1,5_ = 22.78, *P* = 0.0050 in 2014 and 133%, *F*_1,5_ = 27.66, *P* = 0.0033 in 2015) (Fig. 5).

#### Simpson’s Diversity and Evenness

**Fig. 6.**
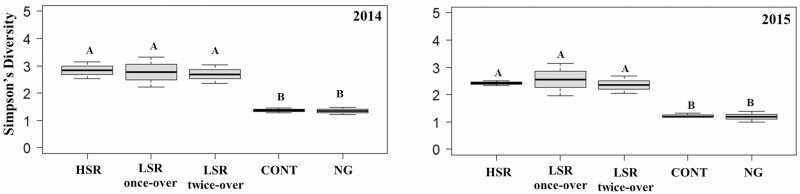
Simpson’s diversity of dung beetles (Coleoptera: Scarabaeoidea) collected at Barta Brothers Ranch in 2014 and 2015. High-stocking rotational grazing (HSR), low-stocking rotational grazing with once-over (LSR once-over) and twice-over (LSR twice-over), continuous grazing (CONT), and no grazing (NG). Letters indicate significance in treatments (*P* < 0.05). Means with the same letters are not significantly different. Vertical bars indicate SEs of the means.

**Fig. 7.**
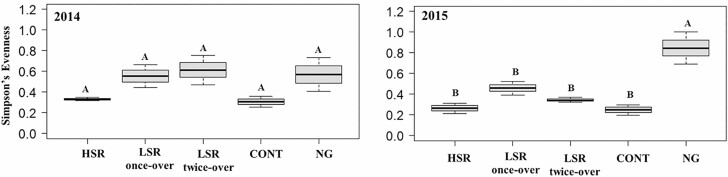
Simpson’s evenness of dung beetles (Coleoptera: Scarabaeoidea) collected at Barta Brothers Ranch in 2014 and 2015. High-stocking rotational grazing (HSR), low-stocking rotational grazing with once-over (LSR once-over) and twice-over (LSR twice-over), continuous grazing (CONT), and no grazing (NG). Letters indicate significance in treatments (*P* < 0.05). Means with the same letters are not significantly different. Vertical bars indicate SEs of the means.

Simpson’s diversity and evenness calculated at the BBR are shown in Figs. 6 and 7, respectively. The Simpson’s diversity indexes were similar for all rotationally grazed treatments in both years (Fig. 6). However, the Simpson’s diversity indexes were significantly lower under the CONT compared to the HSR (52%, *F*_1,5_ = 10.19, *P* = 0.0242 in 2014 and 45% *F*_1,5_ = 7.74, *P* = 0.0388 in 2015), the LSR once-over (51%, *F*_1,5_ = 9.31, *P* = 0.0284 in 2014 and 48%, *F*_1,5_ = 9.42, *P* = 0.0278 in 2015), and the LSR twice-over (49%, *F*_1,5_ = 8.22, *P* = 0.0351 in 2014 and 44%, *F*_1,5_ = 6.91, *P* = 0.0466 in 2015) (Fig. 6). No significant differences were observed between CONT and NG treatments either year (*P* = 0.9702 in 2014 and *P* = 0.9993 in 2015) (Fig. 6). The Simpson’s evenness values were similar among grazing treatments in 2014 (Fig. 7). In 2015, however, the NG had significantly higher evenness compared to the HSR (221%, *F*_1,5_ = 25.60, *P* = 0.0039), LSR once-over (84%, *F*_1,5_ = 11.26, *P* = 0.0202), LSR twice-over (150%, *F*_1,5_ = 19.39, *P* = 0.0070), as well as the CONT (240%, *F*_1,5_ = 26.92, *P* = 0.0035) (Fig. 7).

## Discussion

Preserving and, more importantly, facilitating the increased proliferation of dung beetle populations continue to be areas of ongoing concern among insect ecologists as well as the ranching communities around the world (Barbero et al. 1999, Hutton and Giller 2003, Tonelli et al. 2019). This is due to the ecosystem services that these beetles provide to cattle-grazed rangelands (Nichols et al. 2008, Slade et al. 2016). However, populations of these critical beetles have continued to decline in recent years due to changes in agricultural practices, habitat loss, and insecticide usage (Hutton and Giller 2003, Slade et al. 2016). The dung beetle’s role in preserving ecosystem resilience has made their conservation an increasingly important research agenda (Barbero et al. 1999, Scholtz et al. 2009), especially in context to maintaining healthy rangelands that sustain livestock production.

The total number of dung beetles in our experiment were lower than what was reported in previous studies conducted in Nebraska (Jameson 1989, Whipple and Hoback 2012), and nationwide (Tonelli et al. 2017, Perrina et al. 2020). The total number of dung beetles collected can vary from one study to another depending on soil type, environmental conditions, dung source, vegetation, grazing management practices, and timing and duration of sampling events. For example, Osberg et al. (1994) reported that differences in soil type caused differences in dung beetle abundance, which was attributed to the sensitivity of several species to water holding capacity. Sampling was done when part of the grazing season was dry during late June to September of 2014 and 2015, which may correspond with a period of lower dung beetle activity. Domínguez et al. (2015) reported that dry environments with little rainfall and high temperature can lead to lower total number of dung beetles. A study conducted in Mexico reported that approximately 73% of the total number of dung beetles were captured in October compared to both July and September (Anduaga 2004). Furthermore, the number of dung beetles collected in this study varied between years, which may be due to differences in the duration and time of sampling (Fig. 2). Also, any differences in soil temperature and soil water content may result in differences in the number of dung beetles collected between these 2 yr (Osberg et al. 1994). Previous measurements at the same experimental site in 2014 and 2015 concluded that soil moisture and temperature varied between these 2 yr (Evans et al., 2019a).

The dynamics of a naturally occurring community typically consists of a few common species and a few rare species, with the majority being the moderately abundant species (McCabe 2011). Even though numerous dung beetle species were collected over the duration of this study, our abundance curves reveal that we likely captured only the common and moderately abundant species in the community (Fig. 3). If sampling efforts had been doubled or even tripled, several rare species of dung beetles may have been captured. Our results can be attributed to the small amount of time over which the community was sampled. However, despite this limitation, the data reveal the dominant species within these dung beetle communities. This result proves useful as the dominant species will likely have the largest role in dung decomposition.

It is important to understand that different dung beetle species have varying contributions when it comes to dung decomposition. In general, dung beetles can be categorized into three different guilds based on their nesting behavior. These guilds are endocoprids, paracoprids, and telecoprids, also commonly referred to as dwellers, tunnelers, and rollers (Hanski and Cambefort 1991, Simmons and Ridsdill-Smith 2011). In short, dwellers nest within dung pats, tunnelers nest in burrows in the soil underneath dung pats, and rollers nest in balls of dung that are formed from the dung pats and buried underground some distance away from the original source (Hanski and Cambefort 1991). Species from all three guilds were captured in this study. Tunnelers were the dominant group, with *Onthophagus hecate* (Panzer) and *Onthophagus pennsylvanicus* Harold being among the most abundant species at all three ranches (Table 2). As a result, we can infer that the dung is not only being decomposed on the surface by dwellers, but is also being directly incorporated into the soil through the activity of tunnelers as well as rollers.

This study provides additional evidence that some grazing practices may be favorable for the colonization of dung beetles when compared to others. More specifically, higher peak abundance and species richness of dung beetles under grazed treatment compared to NG treatment at the BBR were reported in both years of this study. These results support past research that grazing abandonment can have negative effects on dung beetle communities due to their dependency on dung for food and habitat (Nichols et al. 2009, Treitler et al. 2017). Similarly, Tonelli et al. (2017) reported that dung beetle abundance was higher for moderate grazing intensity compared to low grazing intensity. However, Correa et al. (2019) reported that cattle grazing with stocking rates of 0.5–1.0 AU/ha for at least 70 yr did not affect the species richness and abundance of dung beetles compared to plots without grazing, suggesting that the effect of grazing on dung beetle communities may vary depending on the location, grazing history and management.

The high peak abundance and species richness of dung beetles associated with rotational grazing at high stocking density (i.e., ~500 AU/ha) observed in this study could be due to an increase in the concentration and dispersal of dung pats throughout the pasture (Richards and Wolton 1976, Whipple 2011). A larger herd and the resulting increase in dung deposition could be the most influential grazing strategy for attracting dung beetles. Since dung beetles are attracted to dung primarily by odor, higher stocking densities may favor their colonization (Dormont et al. 2004). However, Sliwinski et al. (2019) reported that using rotational and continuous grazing systems on private ranches in the Sandhills rangeland did not affect biodiversity (vegetation structure and bird abundance), a reason that was attributed to the fact that the goal of many ranchers using different grazing systems is to maximize beef production, not to increase biodiversity and ecosystem services.

As Verdú et al. (2007) reported, grazing affects vegetation structure, thus influencing the diversity of dung beetles compared to no graze areas. Furthermore, livestock grazing can affect the composition of the vegetation (Alemu et al. 2019), and potentially affect dung beetle diversity. Rotational grazing includes periods with no livestock grazing that can positively impact vegetation variability (Teague et al. 2004). Differences in vegetation type can influence the microclimate surrounding the dung pats; therefore, vegetation diversity can offer different habitats, thereby promoting beetle diversity (Romero-Alcaraz et al. 2000).

The similarity in Simpson’s diversity among our rotational grazing treatments contrasts, with the HSR showing greater abundance and more species richness than the LSR treatments. The relative evenness of species may play a role because Simpson’s evenness appeared lower, although not significant, in the HSR. The relationship between Simpson’s evenness and diversity allows the lower evenness in the HSR to bring the diversity index value closer to that of the LSR treatments (Magurran and McGill 2011). The reasons for higher Simpson’s evenness under NG compared to other treatments are likely due to very low dung beetle abundance and species richness. Research involving a larger sample size is needed to explain the mechanistic reasons for the impact of grazing treatment on Simpson’s evenness.

The results of this study may give ranchers and other pastureland owners valuable insight into how they can graze their livestock and at the same time promote dung beetle populations. Much research has suggested the benefits of dung beetles as they provide multiple essential ecosystem services (Nichols et al. 2008, Slade et al. 2016, Perrin et al. 2020). Furthermore, dung beetle activity can contribute to the suppression of dung-breeding livestock pests, including flies, parasitic nematodes, and protozoa (Byford et al. 1992). By quickly breaking down dung pats, dung beetles can disrupt the life cycles of developing pests and help reduce management costs associated with livestock pests (Bryan 1973; Fincher 1973, 1975, 1981). Conserving dung beetle populations by implementing rotational grazing practices could be advantageous by providing improved rangeland health in many regions including Nebraska (Barbero et al. 1999).

This study contributes useful information to an important knowledge gap regarding the effects of grazing practices on dung beetle communities. It has demonstrated that cattle grazing practices can affect dung beetle activity on rangelands. Rotational grazing, especially when integrated with high stocking density, may help enhance the dung beetle community by promoting abundance as well as species diversity. By implementing rotational grazing practices, dung beetle biodiversity might be strengthened to help build and maintain more sustainable rangeland and grassland ecosystems.

## Supplementary Material

nvaa130_suppl_Supplementary_Tables

## References

[CIT0001] Aarons, S R, C R O’Connor, H M Hosseini, and C J P Gourley. 2009. Dung pads increase pasture production, soil nutrients, and microbial biomass carbon in grazed dairy systems. Nutr Cycling Agroecosyst. 84: 81–92.

[CIT0002] Ahlbrandt, T S, and S G Fryberger. 1980. Geologic and paleoecologic studies of the Nebraska Sand Hills. U.S. Government Printing Office, Washington, DC.

[CIT0003] Alemu, A W, R Kröbel, B G McConkey, and A D Iwaasa. 2019. Effect of increasing species diversity and grazing management on pasture productivity, animal performance, and soil carbon sequestration of re-established pasture in Canadian Prairie. Animals 9: 127. 10.3390/ani9040127 PMC6523940 30934844

[CIT0004] Anduaga, S . 2004. Impact of the activity of dung beetles (Coleoptera: Scarabaeidae: Scarabaeinae) inhabiting pasture land in Durango, Mexico. Environ. Entomol. 33: 1306–1312.

[CIT0005] Bang, H S, J H Lee, O S Kwon, Y E Na, Y S Jang, and W H Kim. 2005. Effects of paracoprid dung beetles (Coleoptera: Scarabaeidae) on the growth of pasture herbage and on the underlying soil. Appl Soil Ecol. 29:165–171.

[CIT0006] Barbero, E, C Palestrini, and A Rolando. 1999. Dung beetle conservation: effects of habitat and resource selection (Coleoptera: Scarabaeidae). J. Insect Conserv. 3: 75–84.

[CIT0007] Bertone, M, W Watson, M Stringham, J Green, S Washburn, and M Hucks. 2004. Dung beetles of central and eastern North Carolina cattle pastures. North Carolina Cooperative Extension, North Carolina State University, Raleigh, NC.

[CIT0008] Bornemissza, G F . 1960. Could dung eating insects improve our pastures. J. Aust. Inst. Agri. Sci. 26: 54–56.

[CIT0009] Bryan, R P . 1973. The effects of dung beetle activity on the numbers of parasitic gastrointestinal helmintic larvae recovered from pasture samples. Aust. J. Agric. Res. 24: 161–168.

[CIT0010] Buse, J, M Šlachta, F X Sladecek, M Pung, T Wagner, and M H Entling. 2015. Relative importance of pasture size and grazing continuity for the long-term conservation of European dung beetles. Biol. Conserv. 187: 112–119.

[CIT0011] Byford, R L, M E Craig, and B L Crosby. 1992. A review of ectoparasites and their effect on cattle production. J. Anim. Sci. 70: 597–602. 1347767 10.2527/1992.702597x

[CIT0012] Colwell, R K . 2013. EstimateS: statistical estimation of species richness and shared species from samples. Version 9. User’s guide and application. (http://purl.oclc.org/estimates) Retrieved 23 October 2020.

[CIT0013] Correa, C M, R F Braga, J Louzada, and R Menéndez. 2019. Dung beetle diversity and functions suggest no major impacts of cattle grazing in the Brazilian Pantanal wetlands. Ecol. Entomol. 44: 524–533.

[CIT0014] de la Motte, L G, O Mamadou, Y Beckers, B Bodson, B Heinesch, and M Aubinet. 2018. Rotational and continuous grazing does not affect the total net ecosystem exchange of a pasture grazed by cattle but modifies CO_2_ exchange dynamics. Agric. Ecosyst. Environ. 253: 157–165.

[CIT0015] Domínguez, D, D Marín-Armijos, and C Ruiz. 2015. Structure of dung beetle communities in an altitudinal gradient of Neotropical dry forest. Neotrop. Entomol. 44: 40–46. 26013011 10.1007/s13744-014-0261-6

[CIT0016] Dormont, L, G Epinat, and J Lumaret. 2004. Trophic preferences mediated by olfactory cues in dung beetles colonizing cattle and horse dung. Environ. Entomol. 33: 370–377.

[CIT0017] Evans, K S, M Mamo, A Wingeyer, W H Schacht, K M Eskridge, J Bradshaw, and D Ginting. 2019a. Dung beetles increase greenhouse gas fluxes from dung pats in a North Temperate Grassland. J. Environ. Qual. 48: 537–548. 31180435 10.2134/jeq2018.03.0111

[CIT0018] Evans, K S, M Mamo, A Wingeyer, W H Schacht, K M Eskridge, J Bradshaw, and D Ginting. 2019b. Soil fauna accelerate dung pat decomposition and nutrient cycling into grassland soil. Rangel. Ecol. Manag. 72: 667–677.

[CIT0019] Filgueiras, B K, L Iannuzzi, and I R Leal. 2011. Habitat fragmentation alters the structure of dung beetle communities in the Atlantic Forest. Biol. Conserv. 144: 362–369.

[CIT0020] Fincher, G T . 1973. Dung beetles as biological control agents for gastrointestinal parasites of livestock. J. Parasitol. 59: 396–399. 4735256

[CIT0021] Fincher, G T . 1975. Effects of dung beetle activity on number of nematode parasites acquired by grazing cattle. J. Parasitol. 61: 759–762. 1165561

[CIT0022] Fincher, G T . 1981. The potential value of dung beetles in pasture ecosystems. J. Ga Entomol. Soc. 16: 316–333.

[CIT0023] Gompert, T . 2009. The power of stock density. *In* Proceedings: Grazing Lands Conservation Initiative’s 4th National Conference on Grazing Lands, Sparks, NV.

[CIT0024] Gotelli, N J . 2008. A primer of ecology. Sinauer Associates, Sunderland, MA.

[CIT0025] Guretzky, J A, M Mamo, W H Schacht, J D Volesky, and A B Wingeyer. 2020. Mob grazing increases trampling but not litter deposition on a Nebraska Sandhills sub-irrigated meadow. Crop Forage Turfgrass Manag. 6: e20047.

[CIT0026] Halffter, G, and E G Matthews. 1966. The natural history of dung beetles of the subfamily Scarabaeinae (Coleoptera, Scarabaeidae). Fol. Entomol. Mexican. 12–14: 1–312.

[CIT0027] Hanski, I, and Y Cambefort. 1991. Dung beetle ecology. Princeton UP, Princeton, NJ.

[CIT0028] Holechek, J L, R D Pieper, and C H Herbel. 2011. Range management: principles and practices, 6th edition. Prentice Hall, Englewood Cliffs, NJ.

[CIT0029] Hutton, S A, and P S Giller. 2003. The effects of the intensification of agriculture on northern temperate dung beetle communities. J. Appl. Ecol. 40: 994–1007.

[CIT0030] Jameson, M L . 1989. Diversity of coprophagous Scarabaeidae (Coleoptera) in grazed versus ungrazed sandhills prairie in Western Nebraska. Trans. Nebraska Acad. Sci. 17: 29–35.

[CIT0031] Larsen, T H, and A Forsyth. 2005. Trap spacing and transect design for dung beetle biodiversity studies. Biotropica 37: 322–325.

[CIT0032] Lee, M C, and R Wall 2006. Cow-dung colonization and decomposition following insect exclusion. Bull. Entomol. Res. 96: 315–322. 16768820 10.1079/ber2006428

[CIT0072] Lindsey, T . 2016. Grazing method effects on forage production, utilization, animal performance and animal activity on Nebraska Sandhills meadow. M.S. thesis, University of Nebraska-Lincoln.

[CIT0033] Losey, J E, and M Vaughan. 2006. The economic value of ecological services provided by insects. BioScience 56: 311–323.

[CIT0034] Magurran, A E, and B J McGill. 2011. Biological diversity: frontiers in measurement and assessment. Oxford UP, Oxford, United Kingdom.

[CIT0035] Manning, P, E M Slade, S A Beynon, and O T Lewis. 2017. Effect of dung beetle species richness and chemical perturbation on multiple ecosystem functions. Ecol. Entomol. 42: 577–586.

[CIT0036] McCabe, D J . 2011. Sampling biological communities. Nat. Educ. Knowl. 3: 63.

[CIT0037] Menéndez, R, P Webb, and K H Orwin. 2016. Complementarity of dung beetle species with different functional behaviours influence dung–soil carbon cycling. Soil Biol. Biochem. 92: 142–148.

[CIT0038] Moir, J L, K C Cameron, H J Di, and U Fertsak. 2010. The spatial coverage of dairy cattle urine patches in an intensively grazed pasture system. J. Agric. Sci. 149: 473–485.

[CIT0039] Nebraska Department of Agriculture . 2016. Nebraska agriculture fact card. (http://www.nda.nebraska.gov) Retrieved 23 October 2020.

[CIT0040] Nichols, E, S Spector, J Louzada, T Larsen, S Amezquita, and M E Favila. 2008. Ecological functions and ecosystem services provided by Scarabaeinae dung beetles. Biol. Conserv. 141: 1461–1474.

[CIT0041] Nichols, E, T A Gardner, C A Peres, and S Spector. 2009. Co-declining mammals and dung beetles: an impending ecological cascade. Oikos 118: 481–487.

[CIT0042] Numa, C, C Rueda, J R Verdú, and E Galante. 2010. Influence of grazing activities on species diversity of dung beetles in Mediterranean pastures. Options Mediterr. 92: 277–280.

[CIT0043] Nunes, C A, R F Braga, F de Moura Resende, F de Siqueira Neves, J R C Figueira, and G W Fernandes. 2018. Linking biodiversity, the environment and ecosystem functioning: ecological functions of dung beetles along a tropical elevational gradient. Ecosystems 21: 1244–1254.

[CIT0045] Osberg, D C, B M Doube, and S A Hanrahan. 1994. Habitat specificity in African dung beetles: the effect of soil type on the survival of dung beetle immatures (Coleoptera Scarabaeidae). Trop. Zool. 7: 1–10.

[CIT0046] Penttilä, A, E M Slade, A Simojoki, T Riutta, K Minkkinen, and T Roslin. 2013. Quantifying beetle-mediated effects on gas fluxes from dung pats. PLoS ONE 8: e71454. 23940758 10.1371/journal.pone.0071454 PMC3737124

[CIT0047] Perrin, W, M Moretti, A Vergnes, D Borcard, and P Jay-Robert. 2020. Response of dung beetle assemblages to grazing intensity in two distinct bioclimatic contexts. Agric. Ecosyst. Environ. 289: 106740.

[CIT0048] Ratcliffe, B C . 2013. The dung-and carrion-feeding scarabs (Coleoptera: Scarabaeoidea) of an Amazonian blackwater rainforest: results of a continuous, 56-week, baited-pitfall trap study. Coleopt. Bull. 67: 481–520.

[CIT0049] Ratcliffe, B C, and M J Paulsen. 2008. The Scarabaeoid beetles of Nebraska (Coleoptera: Scarabaeoidea). University of Nebraska State Museum, Lincoln, NE.

[CIT0050] Richards, I R, and K M Wolton. 1976. The spatial distribution of excreta under intensive cattle grazing. Grass Forage Sci. 31: 89–92.

[CIT0051] Romero-Alcaraz, E, and J M Ávila. 2000. Effect of elevation and type of habitat on the abundance and diversity of Scarabaeoid dung beetle (Scarabaeoidea) assemblages in a Mediterranean area from Southern Iberian Peninsula. Zool. Stud. Taipei 39: 351–359.

[CIT0052] Rundquist, D C . 1983. Wetland inventories of Nebraska’s Sandhills. Resource Report No. 9, Nebraska Remote Sensing Center, Conservation and Survey Division, Institute of Agriculture and Natural Resources, The University of Nebraska-Lincoln.

[CIT0053] Santos-Heredia, C, E Andresen, D A Zárate, and F Escobar. 2018. Dung beetles and their ecological functions in three agroforestry systems in the Lacandona rainforest of Mexico. Biodivers. Conserv. 27: 2379–2394.

[CIT0054] SAS/STAT . 2013. User manual, version 9.4. SAS Institute, Cary, NC.

[CIT0055] Scholtz, C H, A L V Davis, and U Kryger. 2009. Evolutionary biology and conservation of dung beetles. Pensoft Publishers, Sofia, Bulgaria.

[CIT0056] Schreiber, E T, J B Campbell, D J Boxler, J J Petersen. 1987. Comparison of beetles collected from the dung of cattle untreated and treated with fenvalerate ear tags and pastured on two range types in Western Nebraska. Environ. Entomol.16: 1135–1140.

[CIT0057] Simmons, L W, and J Ridsdill-Smith. 2011. Ecology and evolution of dung beetles. Wiley-Blackwell, Chichester, United Kingdom.

[CIT0058] Slade, E M, T Riutta, T Roslin, and H L Tuomisto. 2016. The role of dung beetles in reducing greenhouse gas emissions from cattle farming. Sci. Rep. 6: 1–8. 26728164 10.1038/srep18140 PMC4700445

[CIT0059] Sliwinski, M, L Powell, and W Schacht. 2019. Grazing systems do not affect bird habitat on a sandhills landscape. Rangeland Ecol. Manage. 72: 136–144.

[CIT0060] Spaak, J W, J M Baert, D J Baird, N Eisenhauer, L Maltby, F Pomati, V Radchuk, J R Rohr, P J Van den Brink, and F De Laender. 2017. Shifts of community composition and population density substantially affect ecosystem function despite invariant richness. Ecol. Lett. 20: 1315–1324. 28921860 10.1111/ele.12828

[CIT0061] Teague, W R, S L Dowhower, and J A Waggoner. 2004. Drought and grazing patch dynamics under different grazing management. J. Arid Environ. 58: 97.

[CIT0062] Thomas, H S . 2012. HSR grazing: a way to improve pastureland. Countryside Small Stock J. 96: 63–65.

[CIT0063] Tonelli, M, J R Verdú, and M E Zunino. 2017. Effects of grazing intensity and the use of veterinary medical products on dung beetle biodiversity in the sub-mountainous landscape of Central Italy. PeerJ. 5: e2780. 28097048 10.7717/peerj.2780 PMC5237365

[CIT0064] Tonelli, M, J R Verdú, and M E Zunino. 2019. Grazing abandonment and dung beetle assemblage composition: reproductive behaviour has something to say. Ecol. Indicator. 96: 361–367.

[CIT0065] Treitler, J T, J Buse, G M Carpaneto, S Zerbe, and J Mantilla-Contreras. 2017. Effects of dung-pad conditions and density on coprophagous beetle assemblages in a Mediterranean rangeland. Biodivers. Conserv. 26: 1431–1444.

[CIT0066] Verdú, J R, C E Moreno, G Sánchez-Rojas, C Numa, E Galante, and G Halffter. 2007. Grazing promotes dung beetle diversity in the Xeric landscape of a Mexican biosphere reserve. Biol. Conserv. 140: 308–317.

[CIT0067] Whipple, S D . 2011. Dung beetle ecology: habitat and food preference, hypoxia tolerance, and genetic variability. Ph.D. dissertation, University of Nebraska-Lincoln, Lincoln, NE.

[CIT0068] Whipple, S D, and W W Hoback. 2012. A comparison of dung beetle (Coleoptera: Scarabaeidae) attraction to native and exotic mammal dung. Environ. Entomol. 41: 238–244. 22506995 10.1603/EN11285

[CIT0069] Yamada, D, O Imura, K Shi, and T Shibuya. 2007. Effect of tunneler dung beetles on cattle dung decomposition, soil nutrients and herbage growth. Grassland Sci. 53: 121–129.

[CIT0074] Yoshitoshi, R, N Watanabe, T Yasuda, K Kawamura, S Sakanoue, and J Lim. 2016. Methodology to predict the spatial distribution of cattle dung using manageable factors and a Bayesian approach. Agric. Ecosyst. Environ. 220: 135–141.

[CIT0070] Zhou, Y, P H Gowda, P Wagle, S Ma, JP Neel, V G Kakani, and J L Steiner. 2019. Climate effects on tallgrass prairie responses to continuous and rotational grazing. Agronomy 9: 219.

