# Supplementary material for: Abundance and Diversity of Dung Beetles (Coleoptera: Scarabaeoidea) as Affected by Grazing Management in the Nebraska Sandhills Ecosystem
Source: Environ Entomol. 2020 Nov 13;50(1):222–31. doi: 10.1093/ee/nvaa130 (PMC8223031; doi:10.1093/ee/nvaa130)
Supplement: nvaa130_suppl_Supplementary_Tables [file nvaa130_suppl_supplementary_tables.docx]

**Table S1.** Ranch name, landscape position, and grazing management for Brown County Ranch (BCR) and Rock County Ranch (RCR) in 2014 and 2015.

| **Ranch** | **Location** | **Animals** | **Grazing treatment** |
| --- | --- | --- | --- |
| BCR | Meadow | Each ranch location was grazed by a separate herd for the duration of the grazing season. | Two grazing treatments were applied in a randomized complete block design with no replications.   1. low-stocking rotational grazing (~ 20 AU ha^-1^, LSR). 2. high-stocking rotational grazing (~ 500 AU ha^-1^, HSR).   The LSR was similar to the LSR twice-over at the BBR, but with cattle being rotated to a different paddock every week. The HSR was similar to the HSR at the BBR. |
|  | Upland |  | One grazing treatment was applied in a randomized complete block design with no replications.   1. high-stocking rotational grazing (~ 500 AU ha^-1^, HSR). The HSR was similar to the HSR at the BBR. |
| RCR | Meadow |  | Two grazing treatments were applied in a randomized complete block design in 2014 only with no replications.   1. low-stocking rotational grazing (LSR ~ 20 AU ha^-1^). 2. high-stocking rotational grazing (HSR ~ 500 AU ha^-1^).   Both LSR and HSR were similar to the LSR and HSR at the BCR. |
